# Florbetaben amyloid PET acquisition time: Influence on Centiloids and interpretation

**DOI:** 10.1002/alz.13893

**Published:** 2024-07-04

**Authors:** Emily Johns, Hillary A. Vossler, Christina B. Young, Mackenzie L. Carlson, Joseph R. Winer, Kyan Younes, Jennifer Park, Julia Rathmann‐Bloch, Viktorija Smith, Theresa M. Harrison, Susan Landau, Victor Henderson, Anthony Wagner, Sharon J. Sha, Michael Zeineh, Greg Zaharchuk, Kathleen L. Poston, Guido A. Davidzon, Elizabeth C. Mormino

**Affiliations:** ^1^ Department of Neurology and Neurological Sciences Stanford University School of Medicine Stanford California USA; ^2^ Department of Psychology Stanford University Stanford California USA; ^3^ Helen Wills Neuroscience Institute University of California Berkeley Berkeley California USA; ^4^ Department of Epidemiology and Population Health Stanford University Stanford California USA; ^5^ Stanford University Wu Tsai Neuroscience Institute Stanford California USA; ^6^ Department of Radiology Stanford University School of Medicine Stanford California USA

**Keywords:** Alzheimer's disease, biomarkers, clinical trials, neuroimaging, positron emission tomography

## Abstract

**INTRODUCTION:**

Amyloid positron emission tomography (PET) acquisition timing impacts quantification.

**METHODS:**

In florbetaben (FBB) PET scans of 245 adults with and without cognitive impairment, we investigated the impact of post‐injection acquisition time on Centiloids (CLs) across five reference regions. CL equations for FBB were derived using standard methods, using FBB data collected between 90 and 110 min with paired Pittsburgh compound B data. Linear mixed models and *t*‐tests evaluated the impact of acquisition time on CL increases.

**RESULTS:**

CL values increased significantly over the scan using the whole cerebellum, cerebellar gray matter, and brainstem as reference regions, particularly in amyloid‐positive individuals. In contrast, CLs based on white matter–containing reference regions decreased across the scan.

**DISCUSSION:**

The quantification of CLs in FBB PET imaging is influenced by both the overall scan acquisition time and the choice of reference region. Standardized acquisition protocols or the application of acquisition time–specific CL equations should be implemented in clinical protocols.

**Highlights:**

- Acquisition timing affects florbetaben positron emission tomography (PET) scan quantification, especially in amyloid‐positive participants.
- The impact of acquisition timing on quantification varies across common reference regions.
- Consistent acquisitions and/or appropriate post‐injection adjustments are needed to ensure comparability of PET data.

## 1 BACKGROUND

Alzheimer's disease (AD) is characterized by the accumulation of the amyloid beta (Aβ) protein into extracellular neuritic plaques.^1^ Positron emission tomography (PET) imaging has enabled in vivo measurement of amyloid pathology^2^ and consequently has emerged as a diagnostic tool to clarify underlying etiology in patients with dementia,^3^ becoming pivotal in research criteria for biological staging of AD.^4^, ^5^ Amyloid PET is often employed in both clinical trials^6^, ^7^, ^8^, ^9^ and real‐world settings^3^ to assess amyloid positivity. Ensuring consistent and accurate quantification of amyloid PET is crucial for reliable results. Multiple harmonization methods have been proposed to quantify amyloid PET magnitude on a common scale.^10^, ^11^, ^12^, ^13^ The Centiloid (CL) scale is the most widely used approach and focuses on a standardized scale to measure amyloid burden that accounts for differences in amyloid PET ligand and data processing.^10^ These equations are generated by leveraging publicly available paired data sets that include gold standard Pittsburgh compound B (PIB) images alongside F18 ligands collected in the same individual.^1^, ^10^, ^14^, ^15^, ^16^ Using linear regression equations, these paired data sets enable different amyloid ligands to be transformed into PIB units, and then scaled such that 0 reflects the average uptake in young amyloid negative adults and 100 reflects the average uptake in amyloid positive patients with AD dementia.^10^, ^17^, ^18^

One limitation with standard CL methods is reliance on a single acquisition window in the available paired data sets. There are known signal increases over the course of a scan, which is problematic, since in the real‐world setting a broad range of acquisition times are allowable according to U.S. Food and Drug Administration (FDA) labels for amyloid PET ligands.^19^, ^20^ For instance, the FDA label for florbetaben (FBB) specifies the collection of a 15–20 min scan acquired anytime during an acquisition window ranging from 45–130 min post‐injection.^21^ In alignment with the FDA label, the Society of Nuclear Medicine and Molecular Imaging recommends acquiring a 20 min scan, 45–130 min after injection of FBB.^22^ It is important to note that this broad allowable range is designated specifically for visual reads and does not apply to quantification of continuous amyloid burden. This approach is in contrast to most current FBB research protocols that implement a 20 min acquisition that spans 90 to 110 min post‐injection,^17^ which is the same acquisition window available in the paired PIB‐FBB data set used to define FBB CLs.^1^ This disconnect between FDA labels and research protocols presents challenges for computing CLs in a clinical setting.

The goal of this study was, therefore, to quantify the extent of how acquisition timing differences impact the quantification of FBB amyloid PET CLs when derived from a CL equation for FBB scans collected 90–110 min post‐injection of radiotracer. We also sought to determine whether these effects vary as a function of amyloid burden and reference region. Although the whole cerebellum is frequently used as a reference region for cross‐sectional quantification of FBB, some longitudinal work promotes the integration of white matter (WM) reference regions for amyloid ligands such as florbetapir^23^ and PIB.^24^ As amyloid PET becomes integrated into clinic, it is important to understand the degree to which acquisition‐time and reference‐region selection impact quantification.

## 2 METHODS

### 2.1 Participants

We analyzed FBB PET data from 245 research participants recruited from the Iqbal Farrukh and Asad Jamal Stanford Alzheimer's Disease Research Center (ADRC; *N* = 175),^25^, ^26^ the Stanford Memory Clinics (*N* = 7), or from two studies of healthy aging (*N* = 26 from the Stanford Aging and Memory Study [SAMS],^27^, ^28^ (*N* = 37) from the Attention, Memory, and Aging Study at Stanford (AMASS), (Table 1). Thus, this study utilized data from separate, ongoing amyloid PET studies at Stanford. Outreach efforts across these studies include community events and newspaper advertisements. Seventy‐eight percent of the total cohort in this study self‐identifies as non‐Hispanic White, which is a limitation of this work. Clinical diagnosis was determined at a clinical consensus meeting by a panel of neurologists and neuropsychologists for all participants, taking into consideration neurological findings, neuropsychological test results, and other clinical data, except for the AMASS study, which used the Montreal Cognitive Assessment (MoCA)^29^ and other neuropsychological test results to confirm clinically unimpaired (CU) status.

**TABLE 1:** Sample demographics.

	Overall (*N* = 245)	Subset (*N* = 118)
**Age**		
Mean (SD)	72.0 (8.12)	72.1 (8.45)
Median [Min, Max]	72.4 [36.7, 95.0]	72.9 [41.4, 95.0]
**Sex**		
Female	130 (53.1%)	60 (50.8%)
Male	115 (46.9%)	58 (49.2%)
**Education**		
Mean (SD)	17.0 (2.41)	16.8 (2.60)
Median [Min, Max]	17.5 [8.00, 25.0]	17.0 [8.00, 22.0]
Missing	17 (6.9%)	14 (11.9%)
**Race**		
White	208 (84.9%)	104 (88.1%)
Asian	22 (9.0%)	8 (6.8%)
American Indian/Alaska Native	2 (0.8%)	2 (1.7%)
Black or African American	1 (0.4%)	0 (0.0%)
More than one race	2 (0.8%)	0 (0.0%)
Native Hawaiian or other Pacific Islander	2 (0.8%)	0 (0.0%)
Unknown/not reported	8 (3.3%)	4 (3.4%)
**Ethnicity**		
Hispanic or Latino	21 (8.6%)	9 (7.6%)
Not Hispanic or Latino	220 (89.8%)	105 (89.0%)
Unknown/not reported	4 (1.6%)	4 (3.4%)
**Clinical status**		
Clinically unimpaired	126 (51.4%)	62 (52.5%)
Mild cognitive impairment	37 (15.1%)	22 (18.6%)
AD dementia	29 (11.8%)	16 (13.6%)
Lewy body spectrum	53 (21.6%)	18 (15.3%)
**Cohort**		
ADRC	175 (71.4%)	77 (65.3%)
AMASS	37 (15.1%)	22 (18.6%)
SAMS	26 (10.6%)	19 (21.7%)
Affiliated clinics	7 (2.9%)	4 (3.4%)

*Note*: The primary analyses incorporated an overall sample of 245 participants, whereas a specific subset of 118 participants, characterized by frame data that spans between 70 and 110 min, was utilized for secondary complementary analyses. The demographic data display age, sex, educational background, race, and ethnicity. Within the “Clinical status” category, AD dementia refers to clinically defined dementia due to Alzheimer's disease and “Lewy body spectrum” denotes participants diagnosed with Parkinson's disease or Lewy body disease. “Cohort” highlights the distinct groups or studies from which participants were recruited: ADRC (Alzheimer's Disease Research Center), AMASS (Attention, Memory, and Aging Study at Stanford), SAMS (Stanford Aging and Memory Study), and affiliated clinics.

All study protocols were approved by the Stanford University Institutional Review Board. Written informed consent was obtained from each study participant or their legally authorized representative.

### 2.2 Amyloid PET acquisition and processing

Amyloid PET scanning with FBB was completed at the Richard M. Lucas Center for Imaging at Stanford University using a PET/MRI (magnetic resonance imaging) scanner (Signa 3T, GE Healthcare). Emission data were collected at minimum between 90 and 110 min following an ≈300 MBq injection of FBB. PET data were reconstructed into 5‐min frames with zero echo time (ZTE) attenuation correction (MRAC).^30^ The average acquisition start time (± SD) was 70.6 ±  9.2 min, resulting in an average of 8 ± 2 5‐min frames per participant.

Data were processed using an MRI‐free pipeline described by Landau et al.^31^ (Figure S1). Native space 5‐min frame dynamic PET data were motion corrected and summed between 90 and 110 min. The 90–110 summed PET file was used to linearly co‐register the frame PET data to the Montreal Neurological Institute (MNI)‐152 T1 template using statistical parametric mapping (SPM)12 (www.fil.ion.ucl.ac.uk/spm). Co‐registered PET data were then warped non‐linearly to a generic amyloid tracer template,^31^ using normalization parameters defined on the 90–110 summed image. Intensity values were extracted from a global target region, as well as from five reference regions: whole cerebellum, cerebellum gray matter (GM), brainstem, subcortical eroded WM, and a composite reference region, composed of the whole cerebellum, brainstem, and eroded WM.^23^, ^31^ Several reference regions were used to examine if the post‐injection timing window had a differential impact on quantification of amyloid burden. Standardized uptake value ratios (SUVrs) were created by dividing the global target region by each of the five reference regions and converted to CLs using reference‐region specific linear equations in accordance with previously described methods^1^, ^10^ (Supplementary Methods 1, Table S1, Figure S2).

**RESEARCH IN CONTEXT**

1. **Systematic review**: Our systematic review of the literature focused on the influence of positron emission tomography (PET) acquisition timing on amyloid PET quantification, which is crucial for both clinical and research purposes in Alzheimer's disease (AD). We evaluated existing studies that employed amyloid PET imaging, particularly with florbetaben, and assessed the methodologies regarding acquisition timing. We also evaluated the use of amyloid PET quantification for participant selection and treatment monitoring in anti‐amyloid clinical trials.
2. **Interpretation**: Our study reveals that post‐injection acquisition timing is a significant determinant of Centiloid (CL) values in amyloid PET scans. We specifically found that CL values increase when using cerebellar and brainstem reference regions but decrease with white matter reference regions. These results imply that current standardization methods may not account for timing variations, which may influence treatment decisions in AD management.
3. **Future directions**: Variations in post‐injection acquisition timing are likely to occur in a real‐world setting. There is a need to integrate appropriate post‐acquisition adjustments to enable accurate quantification of PET data across different acquisition windows.

We evaluated amyloid positivity both based on a traditional dichotomous using a CL threshold of 18 (Aβ–: CL <18; Aβ+: CL ≥18) extracted from the summed 90–110 min post‐injection image using the whole cerebellum reference region.^17^ In addition to the examination of dichotomous amyloid status, participants were assigned to one of four CL bins (<10, 10–25, >25–50, > 50, S. Methods 1, Figures S3 and S4).^32^

### 2.3 Statistical analysis

Data were analyzed using R version 4.2.0. To examine change in CL as a function of post‐injection acquisition time, we used linear mixed models (LMMs) as described below:

1. Centiloid_ij _= β_1_ + β_2_Time_ij_ + β_3_Aβ Status_i_ + [β_4_ Aβ Status_i_ × Time_ij_] + b_1i_+ b_ij_
2. Centiloid_ij _= Mean CL at each 5‐min frame.
3. Time_ij _= Time in 20 min increments, relative to injection and centered at 100 min.
4. Amyloid Status_i _= Aβ− or Aβ+ (defined at CL = 18); or a four‐level variable (<10, 10–25, 25–50, >50 CL).
5. b_1i _= Random intercept for each participant.
6. b_ij _= Random slope for each participant.

Post hoc contrasts were used to compare Aβ as well as CL bin groups. Variability in estimated slopes across individuals was visualized by extracting random slopes from an LMM predicting Centiloid_ij_ with Time_ij_ as the only predictor and plotting these slopes against CL bin.[Table alz13893-tbl-0002]

Secondary analysis was performed in the subset of 118 participants with 5‐min frame data spanning 70–110 min post‐injection. The MRI‐free pipeline was repeated on the 70–90 min summed data, to enable extraction of intensity values from this earlier time window (Figure S1). One‐sample *t*‐tests were used to determine whether CL differences between 70–90 min and 90–110 min were greater than zero, and two‐sample *t*‐tests were used to contrast these differences across Aβ groups. Effect sizes for the difference between Aβ groups was calculated using Cohen's d, where d = (M_2_‐M_1_)/SD_pooled_ with M_2_ and M_1_ representing the mean of each Aβ group and SD_pooled_ being the pooled SD across groups. Bland–Altman plots were used to visualize CL differences as a function of continuous amyloid burden.

## 3 RESULTS

**FIGURE 1:**
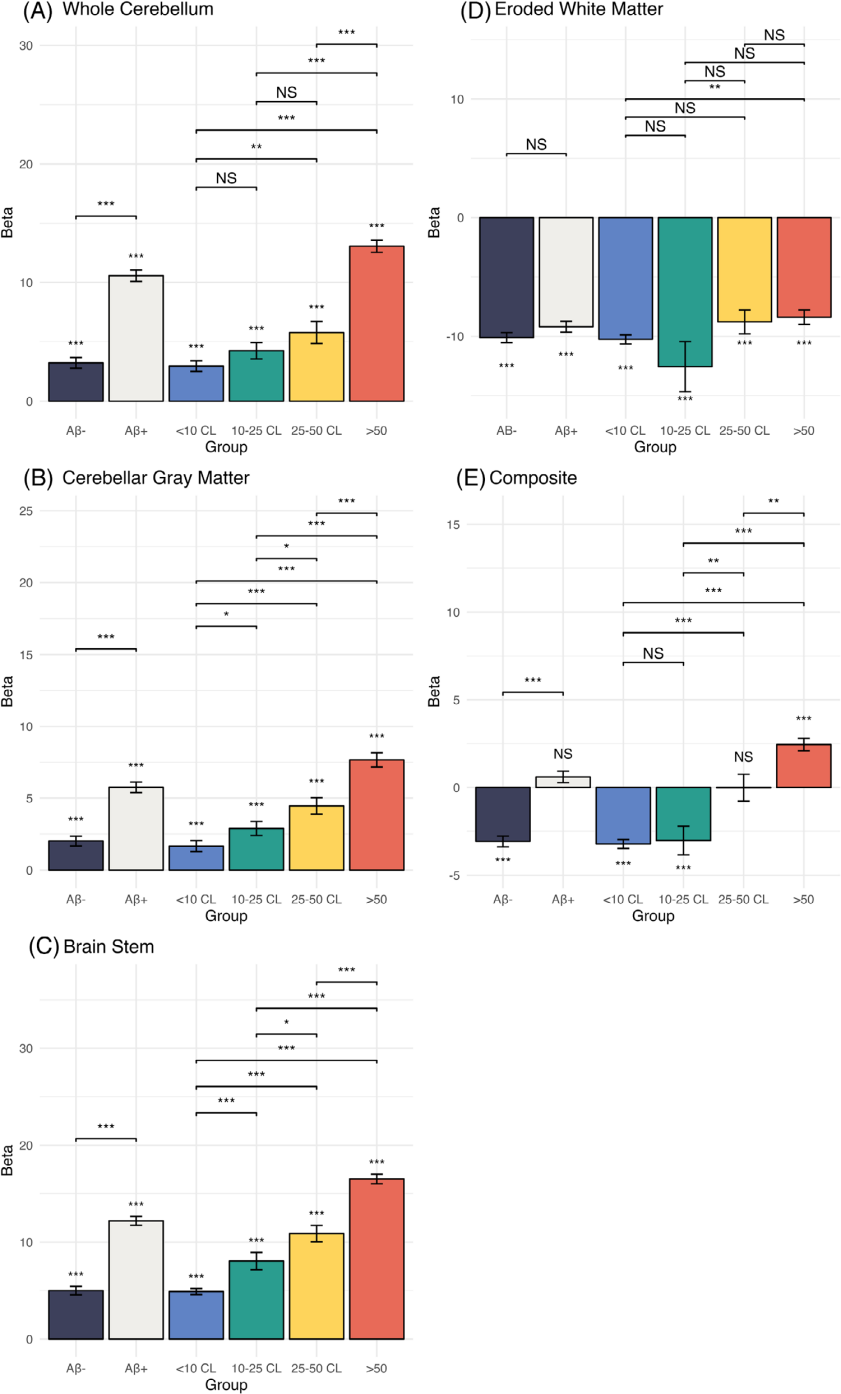
Centiloid (CL) change by amyloid burden and reference region. Model estimates reflect the amount of Centiloid change per 20 min (beta) by dichotomous amyloid status and Centiloid bins for each reference region (A–E). Pairwise statistical significance between groups is represented using “NS” (not significant), ^*^ *p* < 0.05, ^**^ *p* < 0.01, and ^***^ *p* < 0.001.

Using the whole cerebellum reference region, CLs for both Aβ− and Aβ+ groups increased significantly as a function of later acquisition time (Aβ− (*b *± SE): 3.21 ± 0.45, *p* < 0.001; Aβ+ (*b *± SE): 10.56 ± 0.48, *p* < 0.001). The CL increase in the Aβ+ group was statistically greater than that in the Aβ− group (*b *± SE: 7.35 ± 0.66, *p* < 0.001). CLs based on the GM cerebellum and brainstem reference regions showed a similar pattern of increase over acquisition time, whereas the eroded WM and composite reference regions tended to show reductions over time. There were no differences in CL reductions between the Aβ groups for the eroded WM reference region, whereas the Aβ− group showed greater decreases over time than the Aβ+ group for the composite reference region (Table 2; Figure 1).

**TABLE 2 alz13893-tbl-0002:** Linear mixed‐model analysis of Centiloid change by amyloid status and bins.

Global target ratios (Centiloids)					
	Whole cerebellum	Cerebellar gray matter	Brainstem	Eroded white matter	Composite
**Aβ− vs 0**	**3.21 (0.45), <0.001**	**2.02 (0.34), <0.001**	**4.99 (0.44), <0.001**	**−10.11 (0.43), <0.001**	**−3.08 (0.30), <0.001**
**Aβ+ vs 0**	**10.56 (0.48), <0.001**	**5.76 (0.36), <0.001**	**12.19 (0.46), <0.001**	**−9.19 (0.46), <0.001**	0.60 (0.32), 0.06
**Aβ− vs Aβ+**	**7.35 (0.66), <0.001**	**3.74 (0.50), <0.001**	**7.20 (0.64), <0.001**	0.92 (0.63), 0.14	**3.68 (0.45), <0.001**
**<10 CL vs 0**	**2.95 (0.45), <0.001**	**1.67 (0.38), <0.001**	**4.89 (0.31), <0.001**	**−10.27 (0.39), <0.001**	**−3.22 (0.25), <0.001**
**10**–**25 CL vs 0**	**4.24 (0.69), <0.001**	**2.89 (0.49), <0.001**	**8.04 (0.90), <0.001**	**−12.57 (2.11), <0.001**	**−3.03 (0.82), <0.001**
**25**–**50 CL vs 0**	**5.79 (0.93), <0.001**	**4.45 (0.57), <0.001**	**10.88 (0.84), <0.001**	**−8.78 (1.00), <0.001**	−0.02 (0.77), 0.98
**>50 CL vs 0**	**13.07 (0.51), <0.001**	**7.66 (0.49), <0.001**	**16.53 (0.49), <0.001**	**−8.39 (0.61), <0.001**	**2.44 (0.35), <0.001**
**<10 vs 10**–**25 CL**	1.29 (0.82), 0.11	1.22 (0.61), 0.05	**3.15 (0.95), <0.001**	−2.30 (2.14), 0.28	0.19 (0.85), 0.82
**<10 vs 25**–**50 CL**	**2.84 (1.04), 0.01**	**2.78 (0.68), <0.001**	**5.99 (0.89), <0.001**	1.48 (1.08), 0.17	**3.20 (0.81), <0.001**
**<10 vs >50 CL**	**10.12 (0.68), <0.001**	**6.00 (0.62), <0.001**	**11.64 (0.58), <0.001**	**1.88 (0.72), 0.01**	**5.67 (0.43), <0.001**
**10**–**25 vs 25**–**50 CL**	1.54 (1.16), 0.18	**1.56 (0.75), 0.04**	**2.84 (1.23), 0.02**	3.79 (2.33), 0.10	**3.01 (1.12), 0.01**
**10**–**25 vs >50 CL**	**8.82 (0.86), <0.001**	**4.78 (0.69), <0.001**	**8.49 (1.03), <0.001**	4.18 (2.19), 0.06	**5.47 (0.89), <0.001**
**25**–**50 vs >50 CL**	**7.28 (1.07), <0.001**	**3.21 (0.75), <0.001**	**5.65 (0.97), <0.001**	0.40 (1.17), 0.74	**2.46 (0.85), 0.004**

*Note*: Results of a linear mixed‐model analysis, focusing on the variations in Centiloid (CL) change per 20 min across different dichotomous amyloid beta (Aβ) groups and predefined Centiloid bins. The beta estimates provided are unstandardized, accompanied by their standard errors and associated *p*‐values (unstandardized beta (standard error), *p*‐value). The table shows a comparison of Centiloid changes across multiple reference regions, including the whole cerebellum, cerebellar gray matter, brainstem, eroded white matter, and composite.

Bolded text indicates a significant result (p‐value < 0.05).

**FIGURE 2:**
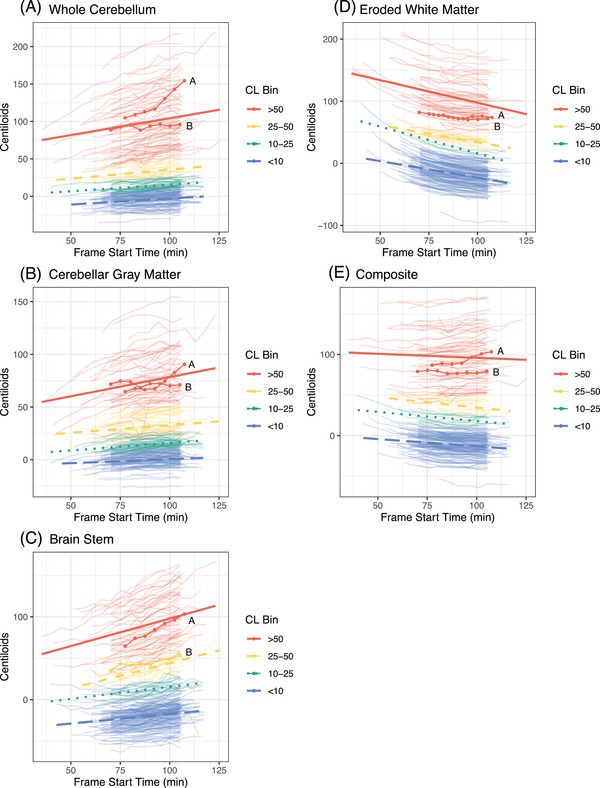
Centiloid (CL) magnitude variation by acquisition time across different reference regions. Individual lines reflect participant level trends for each reference region (A–E). The trend lines in each graph reflect the average progression of centiloids over time. Noticeable patterns and variations can be observed across different brain regions. Two example participants (“A” and “B”) are labeled to emphasize the variability in Centiloid change among individuals with high amyloid burden.

**FIGURE 3:**
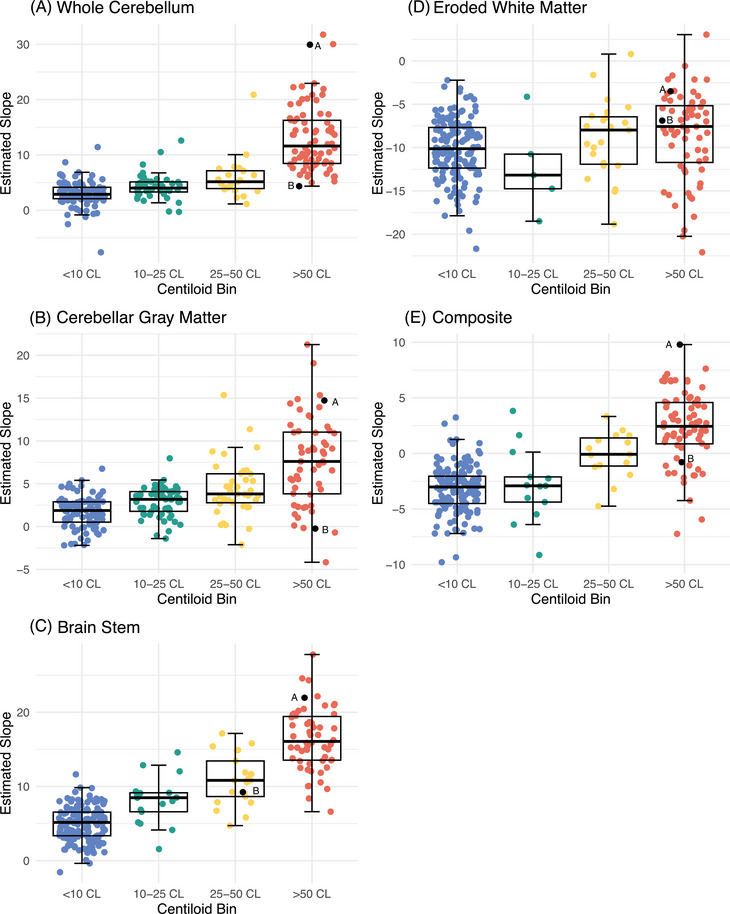
Variability in rate of change across Centiloid bins. Slopes (rate of change over 20 min window) for each participant are plotted across Centiloid bin for each reference region (A–E). Two example participants (“A” and “B”) are labeled to emphasize the variability in estimated change among individuals with high amyloid burden.

Similar patterns of CL differences over acquisition time were present when categorizing participants into one of four CL bins (Table 2, Figures 1, 2). Notably, stepwise increases in CLs were observed for the whole cerebellum, with the lowest amyloid burden bin (<10 CL) increasing 2.95 ± 0.45 CL per 20 min, intermediate levels increasing at 4.24 ± 0.69 CL if between 10 and25 CL and 5.79 ± 0.93 CL if between 25 and 50 CL, and the highest bin (>50) increasing 13.07 ± 0.51 CL (Table 2). Examination of estimated increases (Figure 3A–C) and decreases (Figure 3D–E) over time at the individual participant level showed high variability within each CL bin.

**FIGURE 4 alz13893-fig-0004:**
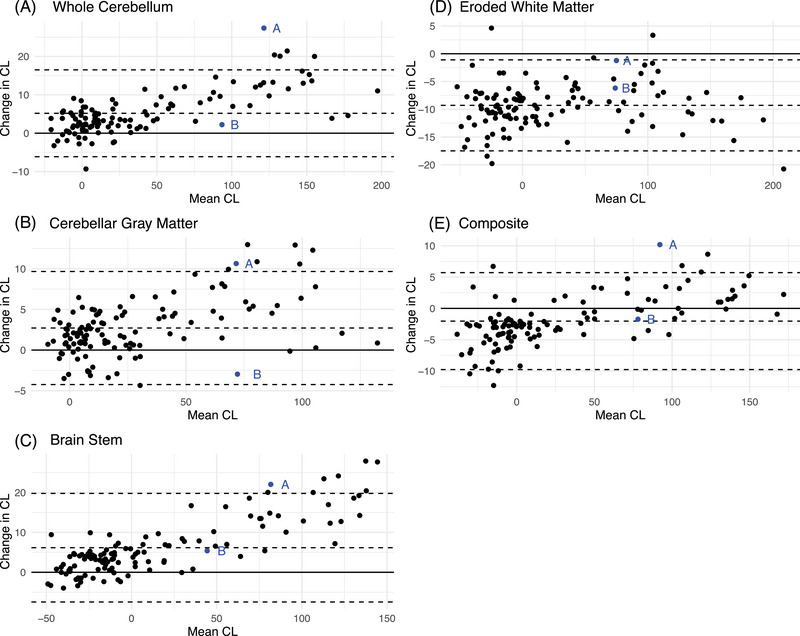
Variability across individuals (using actual data spanning 70–110 min). Bland–Altman plots characterize the difference in Centiloids (CL) for 70–90 and 90–110 min post‐injection summed images for each reference region (A–E). Two example participants (“A” and “B”) are labeled to emphasize the variability in estimated change among individuals with high amyloid burden.

Estimates of CL change were similar when examining the subset of 118 individuals with data spanning 70–110 min post‐injection (Tables 3, 4, Figure 3). For example, CLs using the whole cerebellum were on average 2.21 ± 3.08 CL greater at 90–110 min post‐injection compared to 70–90 min post‐injection for the Aβ− group and an average of 8.26 ± 6.29 CL greater for the Aβ+ group. This analysis also confirmed high participant variability for given CLs (Figure 4).

**TABLE 3:** Subset analysis of Centiloid changes across two time intervals.

Group	Mean Δ Centiloid ± SD	*t*	df	*p*‐value	Cohen's d
a.					
Whole cerebellum					
**Aβ−**	**2.21 ± 3.08**	**5.56**	**59**	**<0.001**	**0.72**
**Aβ+**	**8.26 ± 6.29**	**9.99**	**57**	**<0.001**	**1.31**
**<10 CL**	**1.95 ± 3.28**	**4.16**	**48**	**<0.001**	**0.59**
**10**–**25 CL**	**2.94 ± 2.76**	**4.66**	**18**	**<0.001**	**1.07**
**25**–**50 CL**	**3.64 ± 2.99**	**4.54**	**13**	**<0.001**	**1.21**
**>50 CL**	**11.36 ± 5.63**	**12.12**	**35**	**<0.001**	**2.02**
b.					
Cerebellar gray matter					
**Aβ−**	**1.43 ± 2.27**	**4.87**	**59**	**<0.001**	**0.63**
**Aβ+**	**4.05 ± 4.12**	**7.48**	**57**	**<0.001**	**0.98**
**<10 CL**	**1.34 ± 2.32**	**4.06**	**48**	**<0.001**	**0.58**
**10**–**25 CL**	1.03 ± 3.04	1.47	18	0.16	0.34
**25**–**50 CL**	**2.12 ± 1.98**	**4**	**13**	**0.002**	**1.07**
**>50 CL**	**5.7 ± 3.88**	**8.82**	**35**	**<0.001**	**1.47**
c.					
Brainstem					
**Aβ−**	**2.55 ± 3.18**	**6.21**	**59**	**<0.001**	**0.80**
**Aβ+**	**9.9 ± 7.79**	**9.68**	**57**	**<0.001**	**1.27**
**<10 CL**	**2.33 ± 3.23**	**5.06**	**48**	**<0.001**	**0.72**
**10**–**25 CL**	**2.63 ± 3.04**	**3.78**	**18**	**0.001**	**0.87**
**25**–**50 CL**	**4.58 ± 5.14**	**3.33**	**13**	**0.005**	**0.89**
**>50 CL**	**13.86 ± 6.58**	**12.63**	**35**	**<0.001**	**2.11**
d.					
Eroded white matter					
**Aβ−**	**−9.88 ± 3.99**	**−19.17**	**59**	**<0.001**	**−2.48**
**Aβ+**	**−8.7 ± 4.32**	**−15.32**	**57**	**<0.001**	**−2.01**
**<10 CL**	**−9.94 ± 4.26**	**−16.35**	**48**	**<0.001**	**−2.34**
**10**–**25 CL**	**−10.18 ± 3.33**	**−13.33**	**18**	**<0.001**	**−3.06**
**25**–**50 CL**	**−9.12 ± 2.55**	**−13.37**	**13**	**<0.001**	**−3.57**
**>50 CL**	**−8.01 ± 4.77**	**−10.09**	**35**	**<0.001**	**−1.68**
e.					
Composite					
**Aβ−**	**−3.74 ± 3.07**	**−9.22**	**59**	**<0.001**	**−1.19**
**Aβ+**	−0.3 ± 4	−0.57	57	0.57	−0.07
**<10 CL**	**−3.85 ± 3.34**	**−7.95**	**48**	**<0.001**	**−1.14**
**10**–**25 CL**	**−3.9 ± 2.59**	**−6.35**	**18**	**<0.001**	**−1.46**
**25**–**50 CL**	**−2.43 ± 2.69**	**−3.37**	**13**	**0.01**	**−0.9**
**>50 CL**	**1.54 ± 3.28**	**2.81**	**35**	**0.01**	**0.47**

*Note*: Results of one‐sample *t*‐test analyses evaluating the difference in Centiloids derived from amyloid PET data obtained between two distinct acquisition windows (70–90 min and 90–110 min) for each subject, across reference regions.

Bolded text indicates a significant result (p‐value < 0.05).

**TABLE 4:** Comparative analysis of Centiloid differences across Aβ groups over two time intervals.

Group	*t*	df	*p*‐value	Cohen's d
a.				
Whole cerebellum				
**Aβ− vs Aβ+**	**6.59**	**82.17**	**<0.001**	**1.23**
**<10 vs 10**–**25 CL**	−1.26	38.85	0.21	−0.32
**<10 vs 25**–**50 CL**	−1.82	22.74	0.08	−0.52
**<10 vs >50 CL**	**−8.98**	**52.32**	**<0.001**	**−2.13**
**10**–**25 vs 25**–**50 CL**	−0.68	26.76	0.50	−0.24
**10**–**25 vs >50 CL**	−**7.45**	**52.83**	**<0.001**	−**1.74**
**25**–**50 vs >50 CL**	**−6.27**	**43.05**	**<0.001**	**−1.53**
b.				
Cerebellar gray matter				
**Aβ− vs Aβ+**	**4.26**	**88.05**	**<0.001**	**0.79**
**<10 vs 10**–**25 CL**	0.41	26.55	0.68	0.13
**<10 vs 25**–**50 CL**	−1.24	24.18	0.23	−0.34
**<10 vs >50 CL**	**−6.00**	**53.18**	**<0.001**	**−1.42**
**10**–**25 vs 25**–**50 CL**	−1.25	30.64	0.22	−0.41
**10**–**25 vs >50 CL**	**−4.92**	**45.09**	**<0.001**	**−1.29**
**25**–**50 vs >50 CL**	**−4.29**	**44.16**	**<0.001**	**−1.03**
c.				
Brainstem				
**Aβ− vs Aβ+**	**6.67**	**74.98**	**<0.001**	**1.24**
**<10 vs 10**–**25 CL**	−0.36	34.74	0.72	−0.09
**<10 vs 25**–**50 CL**	−1.55	16.04	0.14	−0.6
**<10 vs >50 CL**	**−9.68**	**47.41**	**<0.001**	**−2.34**
**10**–**25 vs 25**–**50 CL**	−1.26	19.61	0.22	−0.48
**10**–**25 vs >50 CL**	**−8.64**	**52.37**	**<0.001**	**−1.99**
**25‐50 vs > 50 CL**	**−5.27**	**30.26**	**<0.001**	**−1.49**
d.				
Eroded white matter				
**Aβ− vs Aβ+**	1.54	114.5	0.13	0.28
**<10 vs 10**–**25 CL**	0.24	41.75	0.81	0.06
**<10 vs 25**–**50 CL**	−0.9	35.76	0.37	−0.21
**<10 vs >50 CL**	−1.93	70.42	0.06	−0.43
**10**–**25 vs 25**–**50 CL**	−1.04	30.93	0.31	−0.35
**10**–**25 vs >50 CL**	−1.97	48.69	0.05	−0.50
**25**–**50 vs >50 CL**	−1.06	42.88	0.30	−0.26
e.				
Composite				
**Aβ− vs Aβ+**	**5.15**	**107.64**	**<0.001**	**0.95**
**<10 vs 10**–**25 CL**	0.07	41.27	0.94	0.02
**<10 vs 25**–**50 CL**	−1.61	25.8	0.12	−0.43
**<10 vs >50 CL**	**−7.36**	**76.65**	**<0.001**	**−1.61**
**10**–**25 vs 25**–**50 CL**	−1.53	27.98	0.14	−0.54
**10**–**25 vs >50 CL**	**−6.6**	**43.9**	**<0.001**	**−1.75**
**25**–**50 vs >50 CL**	**−4.39**	**28.77**	**<0.001**	**−1.27**

*Note*: Outcomes of two‐sample *t*‐tests that explore the variability in Centiloid measurements taken from amyloid PET scans across two time frames (70–90 min and 90–110 min) for each subject, across reference regions.

Bolded text indicates a significant result (*p*‐value < 0.05). t, t value; df, degrees of freedom.

Finally, we explored whether implementing an acquisition‐timing adjustment across participants would enable data collected during an earlier acquisition to be adjusted to reflect a later acquisition. We explored this type of adjustment by first computing the regression equation between values extracted 70–90 min post‐injection to values extracted 90–110 min post‐injection in a subset of 59 participants and then applied that adjustment equation to an independent sample of 59 participants (Supplementary Methods 2, Table S2, Figure S5). Although this adjustment removed group differences between the two acquisition times (Table S3), considerable variability between adjusted and unadjusted values remained across individuals (Figure S6).

## 4 DISCUSSION

The current study characterizes quantitative measures of amyloid PET burden in relation to post‐injection acquisition time. We found that both the Aβ+ and Aβ− groups exhibited increased CLs over time for the cerebellum and brainstem reference regions. However, decreases over time were generally seen when utilizing reference regions that integrated subcortical WM. In addition, increases over time using the cerebellum and brainstem were most pronounced in participants with the highest amyloid burden. These findings underscore the importance of maintaining consistent post‐injection acquisition‐timing protocols and/or adjusting for time‐acquisition differences when quantifying amyloid PET burden.

Our study assessed the impact of reference region on CLs over the course of the scan. The whole cerebellum and cerebellar GM are the most commonly used regions, as they are known to show limited Aβ plaque deposition and have blood flow characteristics similar to those of the target regions.^33^ However, florbetaben (or FBB) is known to have off‐target/non‐specific binding in the WM, which could explain the differences in trends observed between the cerebellum regions, brainstem, and eroded WM region.^34^ Alternative reference regions incorporating WM have been shown to enhance sensitivity for detecting a change in amyloid PET burden over time.^23^, ^24^, ^35^ For instance, including WM in a composite reference region reduced the coefficient of variation when examining rates of change in florbetapir amyloid signal over 2 years compared to using cerebellum reference regions.^23^ This increased sensitivity may be attributable to controlling for sources of within‐participant noise related to off‐target binding and/or partial volume effects. However, it is essential to note that this conclusion is specific to florbetapir scans and may not generalize to other radiotracers. A study focusing on FBB scans endorsed using cerebellar GM and the whole cerebellum as optimal reference regions for tracking longitudinal changes in FBB PET scans.^33^ Our study, which focuses on tracer dynamics within a single scanning session as opposed to the longitudinal amyloid PET change over years, provides additional evidence that cerebellar versus WM reference regions will differentially impact the quantification of amyloid burden. Given that longitudinal amyloid PET may be used to assess anti‐amyloid therapeutics over time within an individual patient, it will be necessary to explore further the impact of reference region selection on quantification in this specific clinical context.

The present findings[Fig alz13893-fig-0004] of this study have immediate and clinically relevant implications. Amyloid PET scans are widely utilized as a diagnostic tool to confirm underlying pathology and monitor disease progression over time in observational and clinical trial research. Although current FDA labels for amyloid ligands do not endorse quantification of amyloid PET values and instead only include qualitative visual reads, quantification is increasingly used in clinical trials for confirming eligibility, and in some cases to determine when an anti‐amyloid therapeutic should be halted after sufficient amyloid clearance. However, a central observation of our research is the bias introduced when a single SUVR‐to‐CL equation—validated using data from 90–110 min post‐injection of FBB—is applied to data acquired outside of this timeframe. Such application can lead to discrepancies, as evidenced by our analyses, which highlight the variances in CL values derived from PET frames not confined to the 90–110 min post‐injection window. It is unclear whether similar approaches of using a single CL equation for various post‐injection time windows will be used in real‐world settings.

Inaccurate quantification may be especially problematic in the context of primary and secondary prevention strategies that target Aβ+ CU individuals, since these individuals have an overall amyloid burden that is lower than Aβ+ patients with clinical impairment. For instance, the mean CL value for Aβ+ patients with mild cognitive impairment or mild AD dementia in the lecanamab trial was 78,^6^ whereas the primary prevention A3 arm of the ongoing AHEAD345 study focuses on CU individuals who demonstrate intermediate amyloid levels ranging from 20 to 40 CL.^36^ We found average increases of 4–6 CL per 20 min in our intermediate CL group, highlighting that confounds related to acquisition timing are also relevant in the context of early intervention and may obscure the ability to determine eligibility in this group. This strategy of halting drug administration based on CL reductions was recently taken in the TRAILBLAZER‐ALZ2 study testing donanemab. Specifically, drug administration was switched to placebo for participants whose amyloid PET levels decreased to less than 11 CL on a single scan at 24 or 52 weeks, or if their CL levels were between 11 and 25 on two consecutive PET scans.^6^, ^7^ Inaccurate quantification related to acquisition timing in this context could lead to erroneous decisions regarding drug administration.

It is expected that CL values will increase over the course of a FBB PET scan, which was consistent with our findings. However, we also noted large variability across participants, ranging between ≈5 and 30 CL per 20 min in the Aβ+ group. Likewise, we demonstrated that adjusting for acquisition‐timing differences using a simple constant would be insufficient for determining an individual patient's projected CL burden at a different time window, further highlighting variability in tracer dynamics across different individuals. Given the magnitude of effects related to acquisition timing on amyloid PET quantification, it is crucial to develop feasible solutions that would work in a real‐world clinical setting. One possibility is implementing a clinical scanning protocol that preserves the same acquisition time for a given participant across their repeat amyloid scans. However, this solution may not be feasible or practical in a real‐world nuclear medicine or radiology facility and would also result in shifted CL values across patients collected at different time windows (even if acquisition timing was held constant for a given participant at repeat scans). Another option is to forgo quantitative values and instead rely on qualitative reads, which is in line with current FDA label guidelines but inconsistent with how amyloid PET quantification has been integrated into current clinical trials.

It is possible that acquisition times may also impact qualitative reads, with stronger GM signal at a later compared to earlier acquisition window for a given patient. Other factors, such as inter‐reader and intra‐reader variability, can impact qualitative reads. Another solution would be to integrate dynamic data to compute PET magnitude as a function of post‐injection time at the individual participant level. This approach was implemented by Pontecorvo et al. to adjust for differences in acquisition times when analyzing a data set of flortaucipir tau PET scans from a study that aimed to collect emission data beginning at 80 min post‐injection, but noted variability in actual scanning start times.^37^ This participant‐specific interpolation approach enables all scans to be placed on a scale that reflects signal magnitude at a specific desired post‐injection time. Such an approach is appealing because it does not involve additional data collection, incorporates participant‐specific information regarding signal dynamics, and involves a straightforward computation. Notably, software could be developed to handle this type of data processing and installed at PET scanner consoles to be integrated during data reconstruction.

The present study has several limitations. Our data were collected at a single site on a PET/MRI research grade scanner and processed with a previously validated MRI‐Free pipeline.^31^ It is unclear how differences in PET scanners (model, manufacturer), attenuation correction, reconstruction methods, and/or image processing pipelines may impact the magnitude of CL changes as a function of acquisition window. In addition, our sample lacked diversity, consisting of mostly highly educated White individuals, which obscures the generalizability of the findings. Furthermore, we examined only one of three currently FDA‐approved PET ligands.^38^, ^39^ Finally, we noted high inter‐individual variability in rates of change across acquisition time within each CL group but were unable to address sources of this variability.

To summarize, by examining within‐participant tracer dynamics during FBB PET scans, we found large effects of acquisition time on CL magnitude. These effects varied as a function of overall amyloid burden, and by reference region. The present research underscores the critical need to develop quantification strategies that will accurately capture amyloid PET levels in a real‐world clinical setting for patients undergoing anti‐amyloid therapies.

## CONFLICT OF INTEREST STATEMENT

S.L. received payment or honoraria from Eisai and IMPACT‐AD. A.W. received consulting fees from Columbia University and received payment or honoraria from Vanderbilt University, Oxford University Press, and the Learning and the Brain Conference. C.B.Y. receives funding from New Vision Research. K.L.P. received consulting fees from CuraSen Therapeutics Inc, Biohaven, and Neuron23, and has stock options in Curasen and Amprion. E.C.M. has been a consultant for Eli Lilly, Biogen Idec, Hoffmann‐La Roche Ltd, Janssen, and Alector. M.Z. receives research funds from General Electric (GE). S.J.S. receives research support from Biogen Idec, F Hoffmann‐La Roche Ltd, Genentech, aribio, and Novartis for her role as Investigator in Clinical Trials. S.J.S. was a consultant for Guidepoint Global, Boxer Capital, Eisai, and Cognition Therapeutics, and has received payment or honoraria from Forefront Collaborative and UptoDate. G.Z. receives research support from GE Healthcare, LifeMI, and Stanford AI Laboratory, and has received payment or honoraria from Bracco; G.Z. has equity in Subtle Medical. V.H. receives research support from Health IQ Insurance and has received payment or honoraria from the Institute for Clinical and Economic Review, American Academy of Neurology, and Oregon Health Sciences University. E.J., G.D., H.V., J.P., V.S., K.Y., J.R.B., and G.D. have nothing to disclose. Author disclosures are available in the Supporting Information.

## CONSENT STATEMENT

Written informed consent was obtained from each study participant or their legally authorized representative.

## Supporting information

Supporting Information

Supporting Information
