# Supplementary material for: Florbetaben amyloid PET acquisition time: Influence on Centiloids and interpretation
Source: Alzheimers Dement. 2024 Jul 4;20(8):5299–310. doi: 10.1002/alz.13893 (PMC11350032; doi:10.1002/alz.13893)
Supplement: Supplementary file 1 — Supporting Information [file ALZ-20-5299-s002.docx]

**Supplementary Materials**

**S. Figure 1. PET processing steps.** Following these processing steps, intensity values were extracted from a global target region from the GAAIN atlas (<https://www.gaain.org/centiloid-project>), which includes frontal, temporal, parietal, insular cortices, precuneus, and anterior striatum. Intensity values were also extracted across five reference regions: whole cerebellum, cerebellum gray matter (GM), brainstem, subcortical eroded white matter (WM), and a composite reference region. The composite reference regions is composed of the whole cerebellum, brainstem, and subcortical eroded WM [1]. The cerebellum and brainstem ROIs were from the GAAIN atlas, and the eroded white matter was from the Normalized Probability Desikan-Killiany Atlas (NPDKA) with subsequent 8mm^3^ erosion [2].

**S. Methods 1: Centiloids and Thresholds:** Centiloid (CL) equations for each reference region (**S. Table 1**) were derived using the Level-2 method described by Klunk et al., 2015 (**S. Figure 2**). We downloaded the [F-18] Florbetaben (FBB) Calibration dataset from https://www.gaain.org/centiloid-project which contained 90-110 FBB PET scans from 25 elderly participants and 10 young controls (Step 1a) [3]. We processed these scans using our local MRI-Free Pipeline and extracted the mean intensity values for the global target and reference region, in this example, the whole cerebellum (Step 2b). We proceeded to calculate the Global Target/Whole Cerebellum SUVR (i.e., ^FBB^SUVR_IND_) for these 35 participants (Step 2c). Next, we downloaded the corresponding PiB SUVRs (^PiB-Calc^SUVR_IND_) as calculated by Rowe et al. [3] from the GAAIN CL Project website (Step 3a). We correlated the corresponding PiB SUVRs to our MRI-Free pipeline derived FBB SUVRs producing the a linear equation ^FBB^SUVR_IND_=0.56(^PiB-Calc^SUVR_IND_ )+0.475 (Step 3b) which we simplified to the following ^PiB-Calc^SUVR_IND_ = (^FBB^SUVR_IND_ – 0.475)/0.56 to convert ^FBB^SUVR_IND_ to ^PiB-Calc^SUVR_IND_ (Step 4b). We then used this equation to convert the ^PiB-Calc^SUVR_IND_ to ^PiB-Calc^CL using the anchor points of 1.01 (PiB SUVR from 34 YC equivalent to 0 CL) and 2.076 (PiB SUVR from 45 AD equivalent to 100 CL) as described in section 2.2.31 by Klunk et al. [4] (Step 4b) by substituting (^FBB^SUVR_IND_ – 0.475)/0.56 for ^PiB-Calc^SUVR_IND_ in 100(^PiB-Calc^SUVR_IND_ – 1.01)/(2.076-1.01) (Step 4c). Simplifying this equation yields the CL equation to convert FBB SUVRs derived using our MRI-Free pipeline to Centiloids (Step 5a). This process was repeated, beginning with Step 2b (extracting and calculating the ^FBB^SUVR_IND_) for each desired reference region.

We examined the impact of using the whole cerebellum as a reference region for determining centiloid thresholds on the consistency of group assignment compared to other reference regions. First, we established linear relationships between CL values calculated using the whole cerebellum (derived from the summed 90-110 minute post-injection scan) and those obtained with the four alternative reference regions (cerebellum gray matter, eroded subcortical white matter, brainstem, and the composite reference region). This relationship allowed us to translate the established whole cerebellum CL threshold of 18 to corresponding thresholds for each of the alternative regions (**S. Figure 3**). By applying these region-specific thresholds, we found group membership remained consistent, with only an average of 2.6% of cases switching their dichotomous Amyloid Status (**S. Figure 4**). As a result of this consistency, we chose to adopt the CL cut-off value of 18, originally set for the whole cerebellum reference region, for all reference regions. Bin assignment into the four CL bins (<10, 10-25, 25-50, >50 CL) was based on the CL value derived from the summed 90-110 minutes post-injection scan for each reference region as we saw more frequent changes in group membership between reference regions (**S. Figure 4**).

**S. Table 1.** CL equation (based on 90-110 minute post-injection acquisition time scans) to convert the SUVR of each reference region to CL.

|  | Global Target | | | | |
| --- | --- | --- | --- | --- | --- |
| Reference Region | **Whole Cerebellum** | **Cerebellar GM** | **Brain Stem** | **Eroded WM** | **Composite** |
| CL Equation | 167.437x SUVR_FBB_- 174.188 | 80.832x SUVR_FBB_- 102.601 | 245.457x SUVR_FBB_- 179.093 | 367.705x SUVR_FBB_- 232.912 | 259.736x SUVR_FBB_- 204.524 |

**S. Figure 2.** Steps to create florbetaben centiloid equation as described by Rowe et al. [3].

**S. Figure 3.** Thresholds for Centiloid (CL) values were determined for each reference region by establishing a linear relationship between CLs calculated using the whole cerebellum as the reference, derived from the summed 90-110 minute post-injection scan (x-axis), and CLs obtained using alternative reference regions (y-axis). Linear equations, as indicated on each plot, were used to transform the CL threshold of 18 for the whole cerebellum reference region to the corresponding thresholds for each alternative reference region.

**
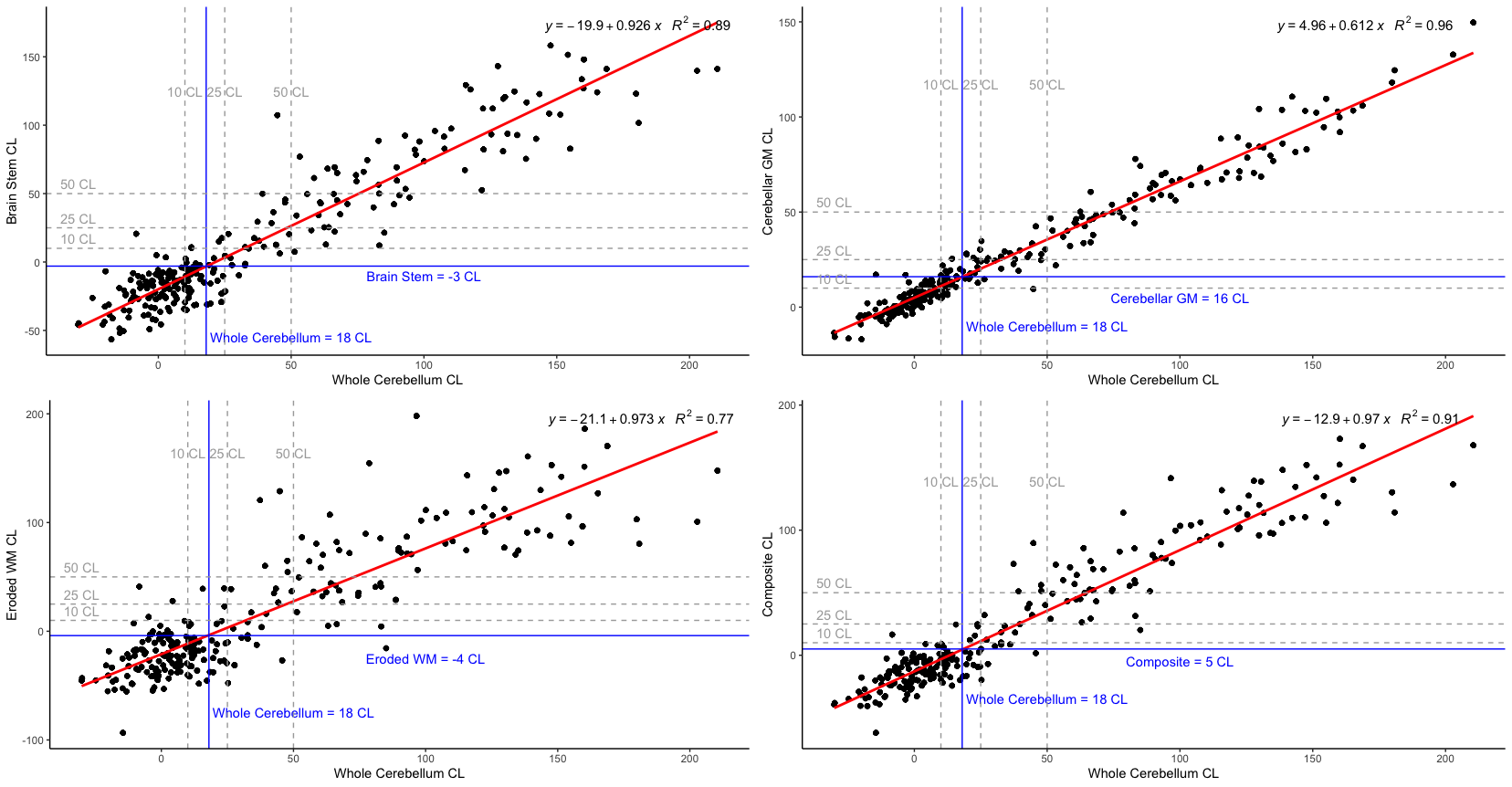
**

**S. Figure 4.** Percentage of participants in each centiloid bin (<10, 10-25, 25-50, >50 centiloids) as determined by the centiloid value derived extracted from the summed 90-110 minutes post-injection image for each of the 5 reference regions examined.

**S. Methods 2.** We developed and applied a linear correction to PET data acquired between 70-90 minutes post-injection based on the relationship between 70-90 and 90-110 data. We divided the remaining 118 subjects into two groups of 59, ensuring equal distribution across each CL Bin. Cohort 1 and Cohort 2 both consisted of 59 subjects each with a 70-90 minute summed PET scan and a 90-110 minute summed PET scan (**S. Table 2**). Cohort 1 was used to develop the linear transformation equations between centiloids derived from 70-90 and 90-110 minute summed PET scan (**S. Figure 5**). The equations derived from Cohort 1 were applied to the 70-90 minute summed PET data of Cohort 2, which we refer to as “adjusted 70-90 minute data.” One sided t-tests were used to characterize the difference between the adjusted 70-90 minute data and 90-110 minute data for Cohort 2 (**S. Table 3**). Bland-Altman plots were used to visualize the individual variability between adjusted 70-90 minute data and 90-110 minute data for Cohort 2 (**S. Figure 6**). Applying a linear correction yielded minimal differences at the group level when comparing the corrected 70-90 to the 90-110 data in Cohort 2. However, the Bland-Altman plots highlighted considerable variability on a subject-by-subject basis, suggesting that individual differences may not be fully captured by a linear model. This indicates the potential need for individual-specific adjustments or alternative modeling approaches to address this variability in future analyses.

**S. Table 2.** Number of subjects in Cohort 1 and Cohort 2.

|  | Centiloid Bin | | | |
| --- | --- | --- | --- | --- |
|  | **<10 CL** | **10-25 CL** | **25-50 CL** | **>50 CL** |
| Cohort 1  (N Subjects) | 25 | 9 | 7 | 18 |
| Cohort 2  (N Subjects) | 24 | 10 | 7 | 18 |

**S. Figure 5.** Relationship between centiloids for each reference region for 70-90 and 90-110 minute summed PET scans in Cohort 1.


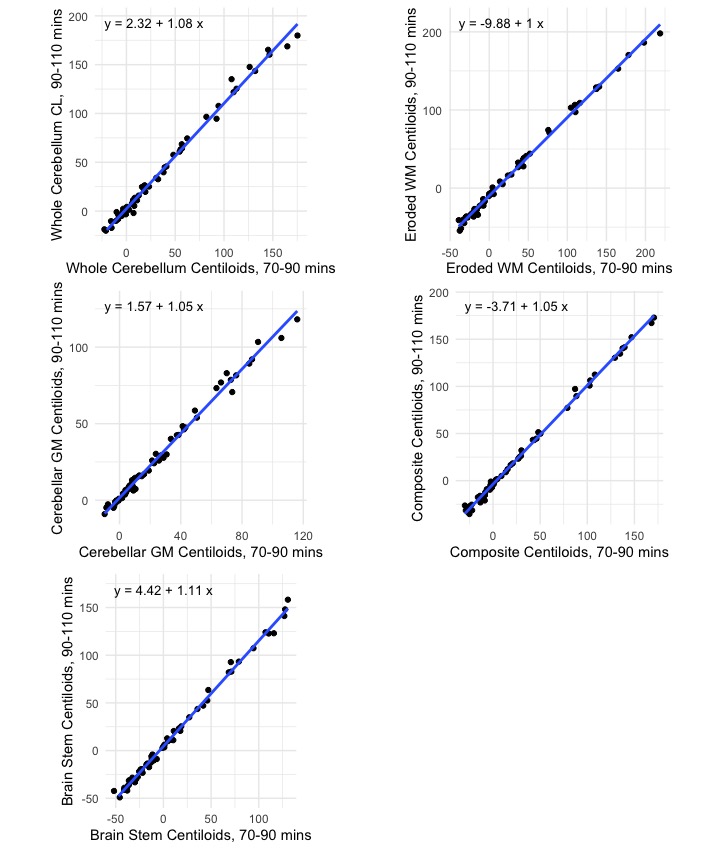


**S. Table 3.** Results of one sided t-tests that characterize the difference between the adjusted 70-90 minute data and 90-110 minute data for Cohort 2.

| Reference region | Delta CL (Mean+SD) | t | df | p-value | Cohen’s D |
| --- | --- | --- | --- | --- | --- |
| Whole Cerebellum | 0.029 + 3.384 | 0.07 | 58 | 0.947 | 0.0005 |
| Cerebellar Gray Matter | 0.509 + 3.147 | 1.242 | 58 | 0.219 | 0.0143 |
| Brain Stem | -0.968 + 4.290 | -1.733 | 58 | 0.088 | -0.017 |
| Eroded White Matter | -0.995 + 4.167 | -1.835 | 58 | 0.072 | -0.017 |
| Composite | -0.572 + 3.085 | -1.425 | 58 | 0.159 | -0.011 |

**S. Figure 6.** Bland-Altman plots showing the individual variability between adjusted 70-90 minute centiloid data and 90-110 minute centiloid data for Cohort 2. The x-axis represents the difference in centiloids between each participant’s adjusted 70-90 minute CL value and their 90-110 minute CL value. The y-axis represents the average between these 2 values for each participant.

**
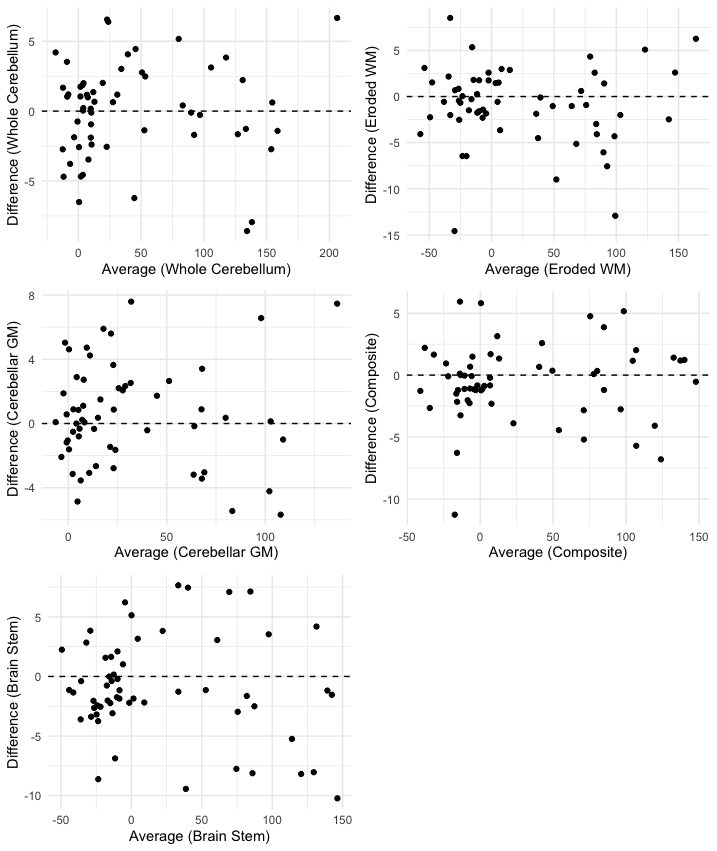
**

**References**

[1] Landau SM, Fero A, Baker SL, Koeppe R, Mintun M, Chen K, et al. Measurement of longitudinal β-amyloid change with 18F-florbetapir PET and standardized uptake value ratios. J Nucl Med Off Publ Soc Nucl Med 2015;56:567–74. https://doi.org/10.2967/jnumed.114.148981.

[2] Landau SM, Ward TJ, Murphy A, Iaccarino L, Harrison TM, La Joie R, et al. Quantification of amyloid beta and tau PET without a structural MRI. Alzheimers Dement 2023;19:444–55. https://doi.org/10.1002/alz.12668.

[3] Rowe CC, Doré V, Jones G, Baxendale D, Mulligan RS, Bullich S, et al. 18F-Florbetaben PET beta-amyloid binding expressed in Centiloids. Eur J Nucl Med Mol Imaging 2017;44:2053–9. https://doi.org/10.1007/s00259-017-3749-6.

[4] Klunk WE, Koeppe RA, Price JC, Benzinger TL, Devous Sr. MD, Jagust WJ, et al. The Centiloid Project: Standardizing quantitative amyloid plaque estimation by PET. Alzheimers Dement 2015;11:1-15.e4. https://doi.org/10.1016/j.jalz.2014.07.003.
